# Computational Modeling and Analysis of Insulin Induced Eukaryotic Translation Initiation

**DOI:** 10.1371/journal.pcbi.1002263

**Published:** 2011-11-10

**Authors:** Joshua Lequieu, Anirikh Chakrabarti, Satyaprakash Nayak, Jeffrey D. Varner

**Affiliations:** School of Chemical and Biomolecular Engineering, Cornell University, Ithaca, New York, United States of America; Stanford University, United States of America

## Abstract

Insulin, the primary hormone regulating the level of glucose in the bloodstream, modulates a variety of cellular and enzymatic processes in normal and diseased cells. Insulin signals are processed by a complex network of biochemical interactions which ultimately induce gene expression programs or other processes such as translation initiation. Surprisingly, despite the wealth of literature on insulin signaling, the relative importance of the components linking insulin with translation initiation remains unclear. We addressed this question by developing and interrogating a family of mathematical models of insulin induced translation initiation. The insulin network was modeled using mass-action kinetics within an ordinary differential equation (ODE) framework. A family of model parameters was estimated, starting from an initial best fit parameter set, using 24 experimental data sets taken from literature. The residual between model simulations and each of the experimental constraints were simultaneously minimized using multiobjective optimization. Interrogation of the model population, using sensitivity and robustness analysis, identified an insulin-dependent switch that controlled translation initiation. Our analysis suggested that without insulin, a balance between the pro-initiation activity of the GTP-binding protein Rheb and anti-initiation activity of PTEN controlled basal initiation. On the other hand, in the presence of insulin a combination of PI3K and Rheb activity controlled inducible initiation, where PI3K was only critical in the presence of insulin. Other well known regulatory mechanisms governing insulin action, for example IRS-1 negative feedback, modulated the relative importance of PI3K and Rheb but did not fundamentally change the signal flow.

## Author Summary

Insulin is a hormone produced by the body that regulates uptake of glucose from the bloodstream. The cellular response to insulin is governed by a complex network of intracellular interactions that ultimately influence cell growth and metabolism. Because of its central role in physiology, insulin signaling has been extensively studied. Yet despite this wealth of research, the relative importance of components in insulin signaling remains unclear. Mechanistic computer simulations have been shown to provide insight into the function of complex systems, such as insulin signaling. In this work we constructed and interrogated a mathematical computer simulation of insulin signaling to better understand the important components of the insulin signaling network. We determined the most important network components and identified network perturbations that can induce dramatic shifts in cellular phenotype. Our results offer an in-depth analysis of the insulin signaling pathway and provide a unique paradigm towards understanding how malfunctions in insulin signaling can result in numerous disease states.

## Introduction

Insulin, the primary hormone regulating the level of glucose in the bloodstream, modulates a variety of cellular and enzymatic processes in normal and diseased cells [1]–[7]. The regulation of cellular function by insulin and insulin-like growth factors I/II (IGF-I/II) is a highly complex process [8]–[14]. Insulin and IGF-I/II interact with insulin receptors (IR), and type I/II IGF receptors (IGF-IR/IIR) in addition to other transmembrane receptors [10]. These interactions ultimately induce gene expression programs or other processes such as translation initiation. Translation rates of many cell cycle and survival proteins are modulated by growth factor, hormone or other mitogenic signals [15]. Insulin induces the activation of class I Phosphoinositide 3-kinases (PI3Ks), which in turn activate the serine/threonine protein kinase Akt and the mammalian target of rapamycin (mTOR). The PI3K/Akt/mTOR signaling axis is important to a variety of cellular programs, including apoptosis [16], cell size control [17] and translation initiation. Among other functions, activation of the PI3K/Akt/mTOR axis results in the phosphorylation of eukaryotic translation initiation factor 4E-binding protein (4E-BPx) family members [18]. Phosphorylation of 4E-BPx causes the release of the eukaryotic translation initiation factor 4E (eIF4E), which is critical to directing ribosomes to the 7-methyl-guanosine cap of eukaryotic mRNAs. Previously, the availability of eIF4E has been shown to be rate limiting for translation initiation in many eukaryotic cell-lines [15], [19]. Given its central role in cell biology, evolutionarily optimized infrastructure like translation might be expected to be robust or highly redundant. Surprisingly, deregulated translation, especially involving growth-factor or insulin induced initiation mechanisms, has been implicated in a spectrum of cancers [20].

Despite the wealth of literature on insulin signaling, the relative importance of the components linking insulin with translation initiation remains unclear. Many investigators have explored this question using both experimental and computational tools. For example, Caron *et al.* recently published a comprehensive map of the mTOR signaling network, including a detailed portrait of insulin induced mTOR activation and its downstream role in translation initiation [21]. Taniguchi *et al.* proposed three criteria to identify the critical nodes of insulin signaling: network divergence, degree of regulation and potential crosstalk [10]. Using these criteria, they identified insulin-receptor (IR), PI3K and Akt as the *critical nodes* of insulin action. Several insightful mathematical models of insulin-signaling have also been published [22]–[25]. While these models vary in their focus and biological scope, none has exclusively focused on how insulin stimulates translation initiation. This particular question was addressed by Nayak *et al.*, who analyzed a family of detailed mathematical models of growth factor and insulin induced translation initiation [26]. Like the Taniguchi *et al.* hypothesis, their study suggested that Akt/mTOR were structurally fragile, and likely the key elements integrating growth factor signaling with translation. However, the Nayak *et al.* model neglected several key features of insulin processing, e.g., negative feedback of IR resulting from mTOR activity.

The objective of this study was to rank-order the importance of components of insulin-induced translation initiation using computational tools. Toward this objective, we analyzed an ensemble of mechanistic mathematical models of insulin induced translation initiation that was a significant extension of our previous work [26]. First, we expanded the original model connectivity to include a detailed description of the regulation and activity of insulin, insulin-like growth factor and platelet-derived growth factor (PDGF) receptor family members (including negative feedback). Second, we refined the description of the phosphorylation state of Akt and its downstream role in the activation of the mTORC1 and mTORC2 complexes. Lastly, we used new model estimation and interrogation techniques to generate and analyze an uncorrelated population of initiation models that were simultaneously consistent with 24 qualitative and quantitative data sets. Interrogation of this model population, using sensitivity and robustness analysis, identified an insulin-dependent switch that controlled translation initiation. Without insulin, a balance between the pro-initiation activity of the GTP-binding protein Rheb and anti-initiation activity of PTEN controlled basal initiation. Rheb knockdown simulations confirmed decreased initiation in the majority of the model population, while translation initiation increased for all models in the population following a PTEN deletion. On the other hand, a combination of PI3K and Rheb activity controlled insulin inducible initiation. PI3K deletion in the presence of insulin removed the ability of the network to process insulin signals, but did not remove initiation altogether. PI3K deletion reduced initiation to approximately 60% of its maximum level. Interestingly, the relative contribution of PI3K versus Rheb to the overall initiation level could be tuned by controlling IRS-1 feedback. In the absence of feedback, PI3K was more important than Rheb to signal propagation, while the opposite was true in the presence of feedback. Taken together, our modeling study supported the Taniguchi *et al.* hypothesis that PI3K was a critical node in the insulin-induced initiation network. However, we also found that the role of PI3K was nuanced; PI3K in combination with Rheb controlled initiation in the presence of insulin, while the combination of PTEN and Rheb controlled basal initiation.

## Results

### Translation initiation model connectivity

**Figure 1:**
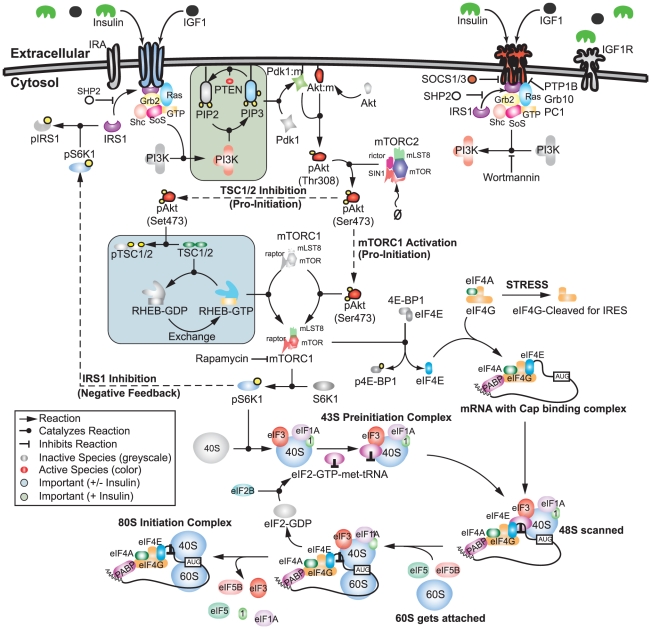
Schematic of the translation initiation signaling network. Growth factors trigger receptor dimerization and the formation of adaptor complexes which activate PI3K. PI3K then signals through PIP2/3 to activate Akt. Activated Akt can then activate mTORC1 either directly or by phosphorylating TSC1/2, an inhibitor of Rheb. Activated mTORC1 can phosphorylate 4EBP1 and activate S6K1, two necessary checkpoints for translation initiation. mTORC1 can also phosphorylate IRS-1, a negative feedback which inhibits formation of the adaptor complex and attenuates insulin signaling.

The translation initiation model consisted of 250 protein, lipid or mRNA species interconnected by 573 interactions (Fig. 1). The model described the integration of insulin and growth-factor signaling with 80S assembly. While other eukaryotic translation initiation mechanisms exist, we focused only on cap-mediated translation as the dominant translation mechanism [27]. The model interactome was taken from literature (SBML file available in the supplemental materials Protocol S1); the connectivity of insulin- and growth-factor induced translation initiation has been extensively studied [14], [28]. The model interactome was not specific to a single cell line. Rather, it was a canonical representation of the pathways involved in insulin and growth-factor induced initiation. Using a canonical network allowed us to explore general features of insulin or growth-factor induced translation initiation without cell line specific artifacts. Binding of insulin or IGF-I/II with IR or IGF-I/IIR promotes the autophosphorylation of the cytosolic domains of these receptors at tyrosine residues. Receptor autophosphorylation promotes the formation of adaptor complexes, which are anchored in place by insulin receptor substrate (IRSx) family members; IRSx are required for the assembly of adaptor complexes involving the SHC-transforming protein 1 (Shc), Son of Sevenless (SoS), growth factor receptor-bound protein 2 (Grb2) and Ras proteins [29]–[31]. In the model we considered only the IRS-1 protein and neglected other IRSx family members. Adaptor complex formation ultimately culminates in the activation of the catalytic subunit of PI3K. Among their many roles, PI3Ks catalyze the phosphorylation of the phospholipid PIP2 to PIP3 [6]. PIP3 is critical to the localization of 3-phosphoinositide-dependent kinase 1 (PDK1) to the membrane, where it phosphorylates the master kinase Akt at Thr308 [32]. Akt is further phosphorylated at Ser473 by the rictor-mammalian target of rapamycin (mTORC2) protein [33]. Once phosphorylated, Akt promotes translation initiation by directly or indirectly activating the mTORC1 protein [1]. Akt directly activates mTORC1 through a novel binding partner known as PRAS40 [34], [35]. However, mTORC1 can also be activated by the GTP bound form of the Ras homologue enriched in brain (Rheb) protein. Without insulin, Rheb is regulated by the tuberous sclerosis complex TSC1/2, which has GTPase activating protein (GAP) activity. Akt directly phosphorylates TSC1/2 which inhibits its GAP activity and allows Rheb-mediated activation of mTORC1 [36], [37]. Activated mTORC1 plays two key roles in translation initiation; first, it activates ribosomal protein S6 kinase beta-1 (S6K1) and second it phosphorylates eukaryotic translation initiation factor 4E-binding protein (4E-BPx) family members [38]. In this study, we included only 4E-BP1 and modeled a single deactivating phosphorylation site. Phosphorylated 4E-BP1 releases eIF4E which, along with other initiation factors, is critical to directing ribosomes to the 7-methyl-guanosine cap structure of eukaryotic mRNAs [28].

Several mechanisms attenuate insulin and growth-factor induced translation initiation. First, insulin signal propagation can be controlled by disrupting adaptor complex formation. For example, we included tyrosine phosphatases and competitive inhibitors such as protein-tyrosine phosphatase 1B (PTP1B), src homology phosphotyrosyl phosphatase 2 (SHP2), growth factor receptor-bound protein 10 (Grb10) and suppressor of cytokine signaling 1/3 (SOCS1/3) which interfere with adaptor complex formation and activity [10], [39]–[41]. Second, several mechanisms control PIP3 formation, PDK1 recruitment and Akt phosphorylation [10]. In the model, we included the phosphatase and tensin homolog (PTEN) protein, which dephosphorylates PIP3 [42], as well as the SH2 (Src homology 2)-containing inositol phosphatase-1 (SHIP1) protein which hydrolyses the 5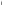-phosphates from PIP3 [43]. Lastly, S6K1 inhibits IRS-1 activity by phosphorylation at Ser318 [44]. S6K1/IRS-1 feedback has been shown to be important in insulin resistance and cancer [14], [45]–[47].

### Estimating an ensemble of translation initiation models using POETs

Translation initiation was modeled using mass-action kinetics within an ordinary differential equation (ODE) framework. ODEs and mass-action kinetics are common methods of modeling biological pathways [48]–[50]. However, ODEs have several important limitations that could be addressed with other model formulations e.g., Partial Differential Equation (PDE) based models. PDEs naturally describe spatially distributed intracellular processes or can be used to model population dynamics using population balance methods [51]. However, the computational burden associated with solving and analyzing systems of PDEs, especially at the scale of the current study, would be substantial. Alternatively, we have addressed both of these ODE shortcomings (without resorting to a PDE formulation) by including well-mixed compartments to account for spatially localized species and processes and have considered an ensemble of models in our analysis to coarse-grain population phenomena. Irregardless of whether we have an ODE or PDE model formulation, both classes of model typically require the identification of a large number of unknown model parameters. The initiation model had 823 unknown parameters (573 kinetic parameters and 250 initial conditions), which were not uniquely identifiable (data not shown). We estimated an experimentally constrained population of parameters using multiobjective optimization. Model parameters were estimated, starting from an initial best fit parameter set, using 24 *in vitro* and *in vivo* data sets taken from literature (Table 1). These training data were taken from multiple independent studies (in different cell lines) exploring insulin and IGF-I/II signaling or *in-vitro* translation initiation. These data were largely western blot measurements of the total or phospho-specific abundance of proteins following the addition of a stimulus or inhibitor. While the use of multiple cell-lines was not ideal, it did allow us to capture a consensus picture of insulin or IGF-I/II initiated signaling (which was useful in understanding the general operational principles of the network). However, one should be careful when applying consensus models to specific cell lines or tissues, as these generally may behave qualitatively differently.

**Table 1:** Objective function list along with species, cell type, cellular compartment, nominal error, training error, prediction error, random error with a randomly generated parameter set and the corresponding literature reference.

O#	Species	Cell Type	Nominal	Training	Prediction	Random	Source
O1	PI3K Activity	3T3-L1 cells	0.01	0.01 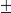 0.00	0.01 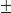 0.00	0.67 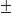 0.20	[82]
O2	PIP3	3T3-L1 cells	0.00	0.00 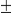 0.00	0.00 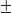 0.00	0.84 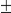 0.08	[82]
O3	pS6K1(T389)	3T3-L1 cells	0.39	0.17 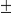 0.15	0.27 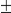 0.24	1.55 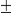 0.49	[35]
O4	pAkt(S473)	3T3-L1 cells	0.38	0.30 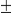 0.23	0.53 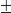 0.29	0.50 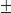 0.38	[35]
O5	IRS1	3T3-L1 cells	0.43	0.47 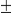 0.62	1.37 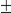 0.71	0.56 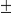 0.58	[35]
O6	pAkt(S473)	393T cells	0.06	0.28 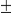 0.32	0.43 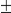 0.35	1.10 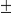 0.31	[35]
O7	pAkt(S473)	C2C12 myotubes	0.05	0.12 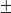 0.13	0.12 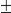 0.13	0.69 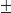 0.11	[83]
O8	pS6K1(T421/S424)	C2C12 myotubes	0.20	0.18 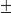 0.07	0.20 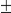 0.10	0.47 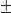 0.22	[83]
O9	pAkt(T308)	HUVEC cells	1.21	0.78 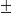 0.38	0.94 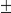 0.36	1.20 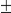 0.79	[84]
O10	IRS-1P(S636/639)	L6 Myotubes	1.34	1.17 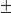 0.37	1.13 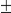 0.35	1.28 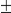 0.38	[53]
O11	pS6K1(T389)	L6 Myotubes	0.98	0.27 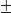 0.33	0.55 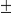 0.64	2.95 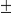 0.51	[53]
O12	pAkt(T308)	L6 Myotubes	0.93	0.62 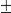 0.36	0.71 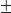 0.34	0.84 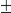 0.48	[53]
O13	IRS-1P(S636/639)	L6 Myotubes	1.24	1.07 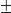 0.38	1.29 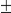 0.31	1.35 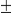 0.36	[53]
O14	pS6K1(T389)	L6 Myotubes	2.36	2.02 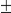 0.43	2.26 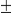 0.24	1.95 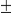 0.38	[53]
O15	pAkt(T308)	L6 Myotubes	0.97	0.39 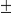 0.35	0.48 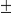 0.33	0.87 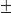 0.82	[53]
O16	pS6K1(T389)	RhoE 3T3 cells	1.33	0.28 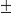 0.33	0.21 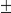 0.25	2.94 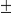 0.54	[54]
O17	c4EBP-P(S65, T37/46)	RhoE 3T3 cells	0.37	0.57 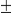 0.33	0.85 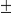 0.38	1.76 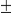 0.43	[54]
O18	Cap-Met-Puro	rabbit reticulocytes	0.46	0.42 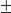 0.46	0.86 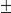 0.73	1.24 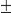 0.71	[55]
O19	43S-mRNA	rabbit reticulocytes	0.19	0.37 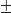 0.39	0.57 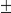 0.47	1.14 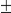 0.64	[55]
O20	pAkt(S473)	A14 NIH 3T3 cells	1.12	0.98 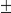 0.23	0.99 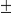 0.23	1.16 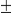 0.15	[56]
O21	pS6K1(T389)	A14 NIH 3T3 cells	1.20	0.57 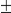 0.29	0.57 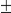 0.23	0.69 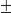 0.21	[56]
O22	Rheb	HeLa cells	0.00	0.15 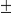 0.83	0.10 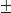 0.71	1.99 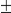 0.09	[56]
O23	pS6K1(T389)	HeLa cells	0.13	0.14 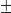 0.11	0.24 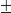 0.23	0.77 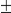 0.58	[56]
O24	c4EBP1-P (T70)	HEK293 cells	0.25	0.34 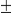 0.26	0.62 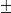 0.41	0.90 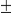 0.22	[56]

The residual between model simulations and each of the experimental constraints was simultaneously minimized using the multiobjective POETs algorithm [52]. We used a leave-three-out cross validation strategy to independently estimate prediction and training error during parameter identification (Table 1). Additionally, a random control (100 random parameter sets) was run to check the training/prediction fitness above random (Table 1). The *training* error for 23 of the 24 objectives was statistically significantly better than the random control at a 95% confidence level. Additionally, for 20 of the 24 objectives, the model *prediction* error was also significantly better than the random control (p
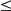
0.05). Of the four remaining objectives (O4,O5,O12 and O13), three involved phosphorylated Akt (O4 and O12) or IRS-1 (O13), each of which had redundant measurements in the objective set that were significant. While the remaining objective, which involved IRS-1 levels (O5), was not significantly better than the random control, the absolute error was small.

**Figure 2:**
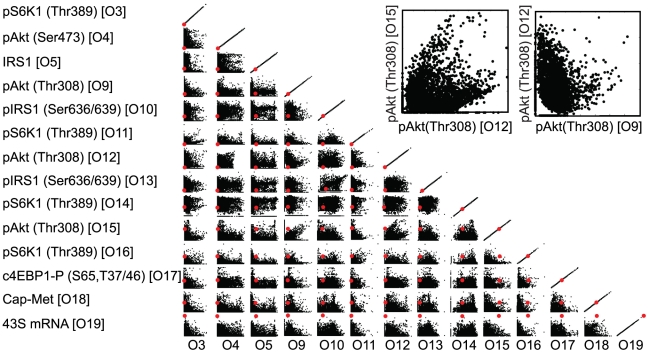
The scaled simulation error (SSE) for selected objective function pairs for N = 5818 rank zero initiation models. The SSEs for objective functions chosen by cross-validation for prediction was set to zero and disregarded when ranking other sets. The red point denotes the performance of the nominal parameter set.

**Figure 3:**
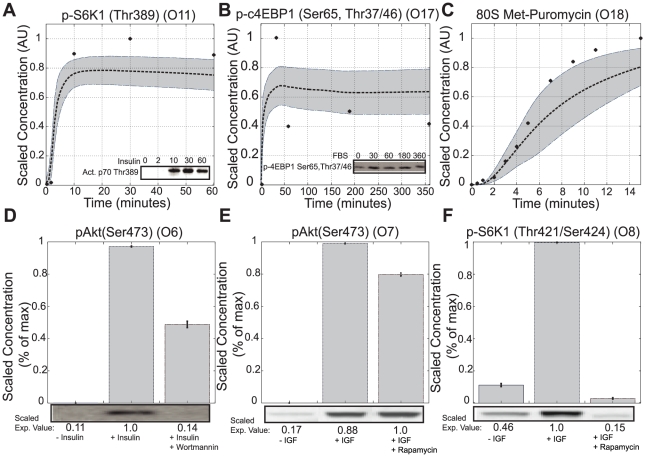
Ensemble performance against selected training objectives (N = 400). Dotted lines represent the simulation mean of the ensemble, while the shaded region denotes the 99.9% confidence estimate for the mean. The solid dots represent the scaled experimental data. **A.** Time course data for p70S6K1 phosphorylation in response to insulin stimulation (L6 Myotubes). **B.** Time course data for c4EBP1 phosphorylation in response to FBS (RhoE 3T3 cells). **C.** In vitro time course of the 80S complex measured by puromycin assay (rabbit reticulocyte). **D.** pAkt(Ser473) levels at 20 minutes in the presence and absence of insulin and wortmannin (393T cells). **E,F.** pAkt(Set473) and activated p70S6K1 levels at 15 minutes in the presence and absence of insulin-like growth factor (IGF) and rapamycin (C2C12 myotubes).

The ensemble of translation models recapitulated diverse training data across multiple cell lines. POETs generated 18,886 probable models with Pareto rank 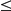
4. Model parameters had coefficients of variation (CV) ranging from 0.65 to 1.10. Further, 89% (512 of 573) of the model parameters were constrained with a CV 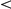
1. The performance of 5,818 rank-zero models is shown in Fig. 2. The majority of objective functions were uncorrelated e.g., O4
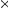
O13 or O12
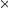
O13 or directly proportional e.g., O3
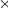
O11 or O9
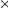
O15. Uncorrelated or proportional objectives suggested the model population simultaneously described each training constraint. However, several other objectives were inversely proportional e.g., O12
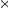
O14. For these pairs, the model was unable to simultaneously fit both training data sets. Surprisingly, these objectives were the same protein pAkt(Thr308) O9
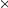
O12 and pS6K1(Thr389) O3
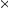
O14, taken from either different cell lines or different labs. This suggested conflicts in the data e.g., cell line variation or differences in specific laboratory protocols, rather than structural inaccuracies in the model, were responsible for the inverse relationship. The key indicators of eukaryotic translation initiation are the phosphorylation of S6K1 and 4E-BP1 [38]. Both Tzatos *et al.* and Villalonga *et al.* performed insightful studies exploring the dynamics of S6K1 and 4E-BP1 phosphorylation in L6 Myotubes and RhoE 3T3 cells [53], [54]. The ensemble recapitulated these observations with error distributions that were statistically significantly better than random parameters (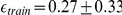, 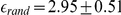; 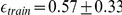, 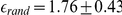) (Fig. 3A and 3B, Table 1). The model population also recapitulated IGF1 induced Akt and S6K1 phosphorylation (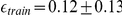, 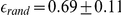; 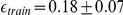, 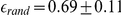) (Fig. 3E and 3F, Table 1). Lorsh *et al.* studied ribosomal assembly dynamics in rabbit reticulocytes, suggesting the formation of the eIF2∶GTP∶Met-tRNA tertiary complex was rate limiting in 80S formation [55]. Our model captured 80S assembly dynamics, including the crucial lag phase in the first two minutes of stimulation (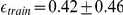, 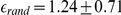) (Fig. 3C, Table 1). Inhibitor data was also used for model training. Without insulin, PI3K was not activated and pAkt (Ser473) levels remained low (Fig. 3D, lane 1). Following insulin stimulation, PI3K activation resulted in increased pAkt(Ser473) levels (Fig. 3D, lane 2). Wortmannin, a PI3K inhibitor, significantly decreased pAkt(Ser473) (Fig. 3D, lane 3). While our model population qualitatively captured this decrease, the levels of pAkt(Ser473) were higher than those observed experimentally. The model was not trained using mTORC1/2 measurements, however species immediately upstream and downstream of mTORC1/2, namely pAkt(Ser473) or S6K1 were used in model training. Without insulin, pAkt(Ser473) and S6K1(Thr421/Ser424) levels were low (Fig. 3E/F, lanes 1). Addition of insulin increased pAkt(Ser473) and S6K1(Thr421/Ser424). Upon rapamycin addition, mTORC1 was inhibited and the levels of phosphorylated S6K1 decreased (Fig. 3E, lane 3). However, because of its position upstream of mTORC1, pAkt(Set473) levels were unchanged (Fig. 3E, lane 3).

**Figure 4:**
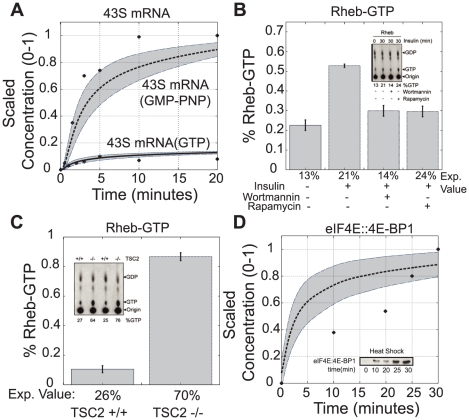
Blind model predictions for the ensemble (N = 400). The predictive ability of model ensemble was assessed by comparing model performance with novel experimental data. Dotted lines represent the simulation mean of the ensemble, while the shaded region denotes the 99.9% confidence estimate for the mean. The solid dots represent the scaled experimental data. **A.** In vitro time course for formation of 43S-mRNA complex. A slowly-hydrolyzable GTP homologue (GMP-PNP) was used in place of GTP to isolate formation of this intermediate complex. GMP-PNP data was used for training while GTP data was used for validation. **B.** Percent of Rheb-GTP to Rheb-GDP in the presence of insulin, wortmannin and rapamycin (A14 NIH 3T3 cells). **C.** Percent of Rheb-GTP to Rheb-GDP in wildtype and TSC2 lacking cells (MEF cells). **D.** 4EBP1 bound EIF4E in the presence of heat shock (CHO.K1 cells).

The model was validated by comparing simulations with *in vivo* and *in vitro* data sets *not* used for training or cross-validation (Table 2). For four of the five prediction data sets, the model demonstrated errors statistically significantly better than a random control (p
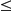
0.05). However, the remaining prediction case (P3), while not significantly different than random, has a small error relative to the other objectives. Data from Lorsh *et al.* was used to validate the dynamics of intermediate ribosomal complexes [55]. The level of 43S mRNA was quantified using both GTP and a non-degradable GTP-like homologue GMP-PNP (Fig. 4A). Data involving GMP-PNP was used for training while data involving GTP was used only for validation (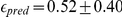, 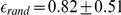). Garami *et al.* explored insulin-induced Rheb activation and the role of TSC1/2 in the presence and absence of wortmannin and rapamycin [56]. We first compared measured versus simulated Rheb-GTP levels, with and without insulin, in the absence of inhibitors. While we captured the qualitative trends, we over-predicted the percentage of GTP bound Rheb (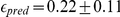, 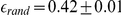) (Fig. 4B). The model also failed to predict sustained Rheb-GTP levels in the presence of rapamycin. This suggested that sustained pAkt(Ser473) levels (observed in Fig. 3E) were not correlated with increased Rheb-GTP activity. Garami *et al.* also measured the levels of GTP bound Rheb in both wild-type and TSC2 knockout cells. Because of TSC2's regulatory role, a TSC2 knockout significantly increased Rheb-GTP levels (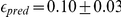, 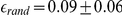) (Fig. 4C). Lastly, the model predicted the levels of 4E-BP1 bound eIF4E in response to heat shock (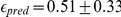, 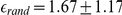) (Fig. 4D) [57]. Because the model was not trained on stress-induced translation inhibition, this result further demonstrated the predictive power of the model population.

**Table 2:** Blind Prediction list along with species, cell type, prediction error, random error with a randomly generated parameter set and the corresponding literature reference.

Prediction#	Species	Cell Type	Compartment	Prediction	Random	Source
P1	43S-mRNA (GTP)	rabbit reticulocytes	in vitro	0.52 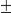 0.40	0.82 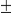 0.51	[55]
P2	Rheb-GTP	A14 NIH 3T3 cells	Total lysate	0.22 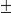 0.11	0.42 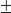 0.01	[56]
P3	Rheb-GTP	A14 NIH 3T3 cells	Total lysate	0.10 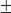 0.03	0.09 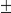 0.06	[56]
P4	eIF4E∶4EBP1	CHO K1 cells	Total lysate	0.51 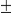 0.33	1.67 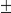 1.17	[57]
P5	pAkt(Ser473)	HEK293 cells	Total lysate	0.27 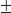 0.09	0.72 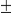 0.09	[56]

### Sensitivity analysis identified robust and fragile features of the initiation architecture

**Figure 5:**
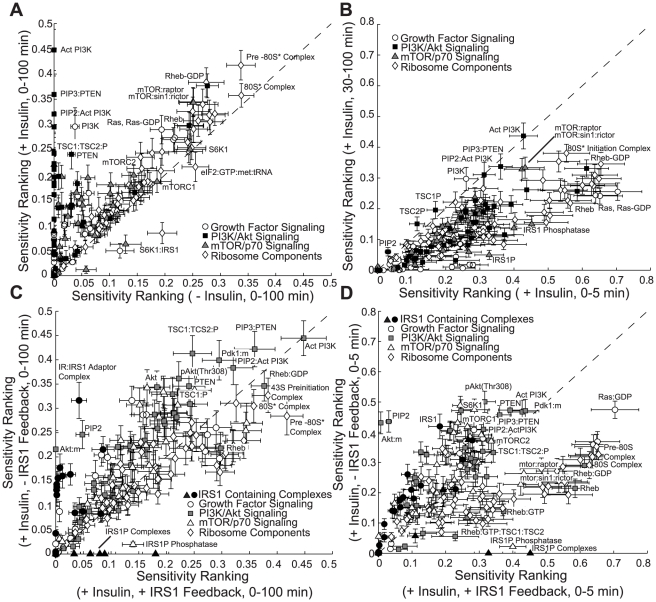
Sensitivity analysis of a population of initiation models (N = 40). Species with a high sensitivity ranking are considered fragile while species with a low sensitivity ranking are considered robust. **A.** Sensitivity ranking of network species in the presence and absence of insulin. **B.** Time-course sensitivity ranking of network species. **C,D.** Sensitivity ranking of network species in the presence and absence of IRS-1 feedback. Black fill denoted complexes containing IRS-1, grey fill denotes PI3K/Akt associated signaling components. Sensitivity values were time averaged over 0–100 minutes and 0–5 minutes, respectively. Error bars denote one standard error in the sensitivity ranking computed over a family of uncorrelated (mean correlation of approximately 0.6) parameter sets selected for the analysis.

Sensitivity analysis generated falsifiable predictions about the fragility or robustness of structural features of the initiation architecture. First order sensitivity coefficients were computed for 40 parameter sets selected from the ensemble (materials and methods), time-averaged and rank-ordered for the 250 species in the model, in the presence and absence of insulin and IRS-1 feedback. The sensitive components of insulin signaling shifted from Rheb in the absence of insulin to a combination of Rheb and PI3K in the presence of insulin. Sensitivity coefficients (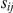) were calculated with and without insulin over the complete 100 min response (Fig. 5A). Globally, processes involved with 80S formation were consistently ranked among the most sensitive, irrespective of insulin. However, the sensitivity of other signal processing components changed with insulin status. For example, without insulin, Rheb/Rheb-GDP were highly fragile (rank
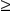
0.25), while PI3K, PIP2, PIP3 and PTEN were highly robust (rank
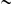
0.0). Surprisingly, the relative sensitivity of these network components changed in the presence of insulin. While the fragility of Rheb/Rheb-GDP shifted modestly upward with insulin, the sensitivity of PI3K and its downstream complexes increased dramatically (rank
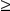
0.45) following insulin stimulation. This suggested that the combination of PI3K and Rheb activity was critical to insulin action over the full 100 min time window. However, it was unclear whether PI3K was always important, or if there was a temporal window in which PI3K became important following insulin stimulation. To explore this question, we time-averaged the sensitivity coefficients over early- and late-phase time periods following insulin stimulation (Fig. 5B). The 0–5 minute time period captured the initial network dynamics, while the 30–100 minute time period captured the network at a quasi-steady state. Generally, network components were more sensitive under dynamic operation (species beneath the 45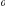 line), compared with steady state. However, there were exceptions to this trend. For example, PI3K, PTEN and TSC1/2 were equally sensitive in both time frames, suggesting these species played important roles in both dynamic and steady state signaling. On the other hand, the Rheb rank decreased from 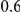 to 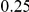 as the network moved toward steady state. Taken together, the sensitivity results suggested that Rheb activity controlled the background level of translation initiation while the PI3K axis in combination with Rheb regulated insulin-induced initiation. Moreover, the transition between PTEN and PI3K control occurred directly after the addition of insulin, giving rise to switch like behavior.

IRS-1 phosphorylation, a well known negative feedback mechanism [14], [45]–[47], attenuated PI3K sensitivity. We explored the role of IRS-1 feedback by comparing sensitivity coefficients under insulin stimulation in the presence and absence of IRS-1 feedback (Fig. 5C). The most significant change without feedback was the sensitivity of the IR∶IRS-1 and adaptor complexes (Fig. 5C, black fill); IR∶IRS-1, which anchors the adaptor complex to the activated receptor and is immediately upstream of PI3K activation, changed from NSS rank 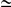
0.04 to 0.32. The sensitivity of the PI3K/Akt signaling axis also increased in the absence of feedback (Fig. 5C, grey fill). Surprisingly, the sensitivity of Rheb and many ribosomal components decreased in the absence of feedback. Similar results were observed when sensitivity coefficients were time averaged over the 0 to 5 min time window (Fig. 5D). These sensitivity calculations suggest that IRS-1 feedback plays a significant role in insulin signaling by modulating the relative importance of PI3K versus Rheb. Thus, IRS-1 feedback though not directly identified as a fragile regulatory motif, has significant effects on network function.

Lastly, the architectural features of the initiation network identified by sensitivity analysis, as either fragile or robust, were likely parameter independent. While first-order sensitivity coefficients are local, we sampled a family of uncorrelated parameter sets (mean correlation of approximately 0.6) to generate a set of consensus conclusions. By sampling over many uncorrelated sets, we calculated how our conclusions changed with different unrelated parameter sets. The distribution of ranking (standard-error shown in Fig. 5) suggested that despite parametric uncertainty, sensitivity analysis over an uncorrelated model population produced a consensus estimate of the strongly fragile or robust elements of the insulin signaling network. Previously, we (and others) have shown that monte-carlo parameter set sampling produced similar results in several studies across many signaling networks [49], [58]–[60].

### Robustness analysis identified key regulators of translation initiation

**Figure 6:**
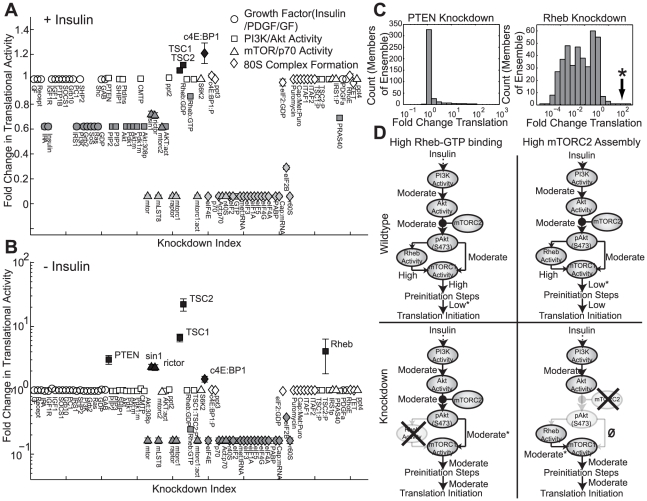
Species knockdown simulations for a population of translation initiation models (N = 400). Simulated knockdowns were performed by removing nodes from the stoichiometric matrix. The relative change in 80S formation resulting from the removal of a species was used to quantify the impact of the knockdown. **A.** Species knockdowns in the presence of insulin. Simulated knockdowns resulted in increased (black), constant (white), moderately decreased (dark grey) or severely decreased (light grey) translational levels. **B.** Species knockouts in the absence of insulin. Simulated knockdowns resulted in increased (black), constant (white), or decreased (grey) translational levels. **C.** Histogram of translation levels across each member of parameter ensemble. Asterisk index indicates parameter sets that were selected for further analysis. **D.** Alternative modes of network operation. For a subset of the ensemble, initiation increased following Rheb or mTORC2 disruption. Asterisk indicates rate-limiting step.

Knockdown simulations were conducted for 92 proteins to estimate the functional connectedness of the initiation network. The effects of the perturbations were quantified by calculating the relative change (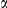) in translational activity (80S formation) for each simulated knockout in the presence (Fig. 6A) and absence (Fig. 6B) of insulin. Knockdown simulations were conducted using 400 models selected from the ensemble based on error and correlation (materials and methods). Proteins were classified based on their impact on translational activity: little or no effect (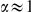, white fill), moderate decrease (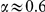, dark grey), critical (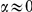, light grey) and increase (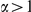, black). Generally, knockdowns in the presence of insulin were more likely to decrease initiation (Fig. 6A). Knockdown analysis identified 24 proteins (or 26% of the network) that were critical to translation initiation irrespective of insulin status; these critical components included mTORC1, S6K1, several initiation factors and other ribosomal components. Sensitivity analysis suggested basal translation was governed by Rheb, while insulin-induced initiation was governed by PI3K. Robustness analysis showed that perturbations in PI3K signaling, in the presence of insulin, restored initiation control to Rheb. Initiation was reduced by 40% by disrupting species immediately upstream or downstream of PI3K; a moderate reduction in the presence of insulin demonstrated that initiation was governed by both PI3K and Rheb. Lastly, deletion of TSC1/2 (negative regulator of Rheb) or 4E-BP1 (sequesters the cap-binding protein eIF4E), increased initiation in the presence of insulin. Interestingly, for several proteins the direction or magnitude of change in initiation activity depended upon the presence or absence of insulin. For example, PTEN deletion significantly increased initiation (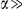
1) in the absence of insulin, but had no effect when insulin was present. On the other hand, PI3K deletion had a moderate reduction on 80S formation in the presence of insulin, but only a small effect in the absence of insulin (Fig. 6B). These results suggested that PI3K and PTEN were conditionally fragile proteins; in the presence of insulin, PI3K is a critical signal processing node, while PTEN acts to restrain inadvertent basal initiation.

Paradoxically, Rheb and mTORC2 subunit (sin1, rictor) knockdowns increased initiation. Our expectation from sensitivity analysis was that a Rheb knockdown would reduce initiation, irrespective of insulin status. However, this was not universally true; some members of the model population showed increased initiation (Fig. 6C). Following the deletion of PTEN, approximately 80% (or 323 of the 400 models sampled) had increased initiation in the absence of insulin. Of these models, 16% (or 51 of 323) had at least a two fold increase in translational activity. This result was expected; deletion of a protein species resulted in a qualitatively similar change in initiation across the ensemble of models. However, for Rheb knockdowns, members of the ensemble demonstrated qualitatively different behavior. For 84% (or 334 of 400) of the models sampled, Rheb knockdowns significantly down-regulated initiation. Thus, the vast majority of models behaved as expected. Interestingly, 20 models (or 5% of the models sampled) had increased translation initiation in the presence of a Rheb knockdown, with 15 models demonstrating greater than a two-fold change (Fig. 6C). Thus, the model population estimated by POETs contained models with qualitatively different behavior. Histograms of sin1 and rictor knockdowns showed a similar trend (results not shown). We explored the flux vectors of these outlying parameter sets to better understand the mechanistic effect of Rheb and rictor/sin1 knockouts. All of the outlying models were in regions of parameter space where the association between Rheb and GTP was very high. Strong Rheb/GTP binding resulted in abnormally high signal flux to mTORC1 despite the inhibitory effects of TSC1/2 (Fig. 6D, top-left). Consequently, less GTP was available for the energy-dependent steps of translation initiation (i.e. formation of eIF2-GTP-met-tRNA tertiary complex). Additionally, strong association between Rheb and GTP resulted in high levels of activated mTORC1 and S6K1. However, despite the high levels of mTORC1, GTP-dependent pre-initiation reactions were rate limiting (Fig. 6D, labeled*). Thus, Rheb knockdown released the network from its GTP limitation and shifted the predominant signaling mode to mTORC2. This shift in signaling, while lowering the activated mTORC1/S6K1 level, ultimately resulted in higher levels of initiation (Fig. 6 bottom-left). On the other hand, the rictor/sin1 knockdown behaved differently. The rate-limiting step for the rictor/sin1 knockdowns was mTORC1 activation: more Rheb-GTP was present than there was mTORC1 to be activated (Fig. 6D top-right). Thus, knockdown of rictor/sin1 prevented the assembly of mTORC2 and freed the mTOR subunit to be used for mTORC1 assembly. This shift toward mTORC1 assembly and activation relieved the Rheb-GTP/mTORC1 bottleneck, resulting in increased initiation.

## Discussion

In this study, we developed and analyzed a population of insulin and growth factor induced translation initiation models. These models described the integration of insulin and growth-factor signals with 80S assembly. A family of model parameters was estimated from 24 transient and steady state data sets using multiobjective optimization. In addition to the training data, the model family also predicted novel data sets not used during model training. The population of initiation models was analyzed using sensitivity and robustness analysis to identify the key components of insulin-induced translation initiation. Without insulin, a balance between the pro-initiation activity of the GTP-binding protein Rheb and anti-initiation activity of PTEN controlled basal initiation. Rheb knockdown simulations confirmed decreased initiation in the majority of the model population. Surprisingly, we also identified a model subpopulation in which deletion of Rheb or mTORC2 components increased initiation. In these cases, removal of Rheb or mTORC2 components relieved a rate-limiting bottleneck e.g., constrained levels of GTP, leading to increased initiation. On the other hand, in the absence of insulin, translation initiation increased for all models in the population following a PTEN deletion. In the presence of insulin, Rheb and PTEN were no longer the dominant arbiters of initiation; a combination of PI3K and Rheb activity controlled inducible initiation, where PI3K was only critical in the presence of insulin. PI3K deletion in the presence of insulin removed the ability of the network to process insulin signals, but did not remove initiation altogether. PI3K deletion reduced initiation to approximately 60% of its maximum level. Interestingly, the relative contribution of PI3K versus Rheb to the overall initiation level could be tuned by IRS-1 feedback. In the absence of feedback, PI3K was more important than Rheb to signal propagation, while the opposite was true in the presence of feedback.

PI3K and PTEN in combination with Rheb are components of a switch that regulates inducible and basal translation initiation. In the absence of insulin, a balance between the pro-initiation activity of Rheb and the anti-initiation activity of PTEN regulated basal initiation. On the other hand, in the presence of insulin, control shifted to a combination of Rheb and PI3K, where PI3K activity regulated the inducible fraction of initiation. Thus, deletion of PTEN, constitutive activation of PI3K or constitutively active Rheb could all induce aberrant translation initiation without an insulin or growth factor signal. Yuan and Cantley noted that every major species in the PI3K pathway is mutated or over-expressed in a wide variety of solid tumors [6]. For example, activating mutations in PIK3CA, the gene encoding the catalytic subunit of PI3K, induces oncogene signaling in colon, brain and gastric cancers [61]. On the other hand, PTEN mutations have long been implicated in a spectrum of cancer types [62]. Both PIK3CA and PTEN mutations induce a pro-initiation operational mode in the absence of growth factor. Likewise, constitutive Rheb activity induces a variety of pleiotropic traits involving translation. For example, Saucedo *et al.* showed that Rheb over-expression in *Drosophila melanogaster* increased cell size, wing area and G1/S cell cycle progression [63]. Rheb and TSC1/2 mutations are also frequently observed in cancer [64], [65]. Taken together, our study supports the supposition of Taniguchi *et al.* that PI3K is a critical arbiter of insulin-induced translation initiation [10]. However, we have also shown that initiation control and particularly the role of PI3K was more nuanced; while insulin or growth-factor inducible initiation was controlled by PI3K, basal initiation was controlled by Rheb. Moreover, in the absence of insulin, PTEN was the critical upstream initiation regulator, not PI3K. This suggested that the relative level of the phosphorylated phospholipids PIP2 and PIP3 was actually the key mediator of initiation. Lastly, Taniguchi *et al.* suggested that Akt was also a key node involved in insulin action. Our previous model directly supports this, however, the current model does not. Rather, our analysis suggested that Rheb was the downstream controller of initiation. These two points of view are not contradictory however, as Rheb activation is driven by phosphorylated Akt.

The initiation model connectivity was assembled from an extensive literature review, however, several potentially important signaling mechanisms were not included. First, we should revisit the role of PRAS40. Currently, PRAS40 acts as a cofactor that aids in pAkt(Ser473)-mediated activation of mTORC1. Sancak *et al* suggested that PRAS40 sequesters mTORC1, and only after phosphorylation by Akt does it releases from mTORC1 [34]. Other groups have also shown that mTORC1 can phosphorylate and inhibit PRAS40, thus providing a positive feedback mechanism for Akt-mediated mTORC1 activation [66], [67]. A more complete description of PRAS40 will enhance our ability to interrogate Akt dependent mTORC1 activation. Second, we need to refine the description of IRS-1 feedback. Currently, we assume a single deactivating phosphorylation event at Ser308. However, several studies have shown that IRS-1 can be phosphorylated at multiple serine sites, which are both activating and deactivating [44], [68]. Additionally, PTEN is known to dephosphorylate activated PDGF receptors and attenuate their activity, a feature not included currently [69]. A more complete description of IRS-1 phosphorylation could help define how, and under what conditions, IRS-1 regulation attenuates PI3K activation. Third, we modeled the regulation of 4E-BPx as a single phosphorylation event where phosphorylated 4E-BPx was unable to bind to eIF4E. In reality, 4E-BPx family members, such as 4E-BP1, have several phosphorylation sites [70] and the release of eIF4E is driven only after multiple conserved phosphorylation events [71]. Additionally, eIF4E can itself be phosphorylated at Ser209; while there is agreement that the phosphorylation of eIF4E does have a regulatory significance, the data is contradictory as to whether it is positive or negative [72]. Fourth, signaling downstream of mTORC1 has also been shown to mediate translation modes beyond those included in our model. eIF3 has been identified as a scaffolding protein that recruits mTORC1 to untranslated mRNA and facilitates S6K1 and 4E-BP1 phosphorylation [73]. S6K1 can also activate eIF4B, a protein that helps eIF4A to unwind the secondary structure of untranslated mRNA [74]. Further, a recently discovered scaffold protein, SKAR, has been shown to assist S6K1 recruitment to mRNA [75]. Lastly, because of mTORC1's unique cellular role, it would be interesting to explore how other aspects of metabolism interact with insulin signaling to mediate decisions between translation, lipid synthesis or proliferation. In these studies, one could imagine constructing *in-vivo* mouse models to explore the physiological role of mTORC1 signaling in important diseases such as diabetes or cancer.

## Materials and Methods

### Formulation and solution of the model equations

The translation initiation model was formulated as a set of coupled non-linear ordinary differential equations (ODEs):
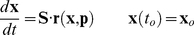
(1)The symbol 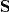 denotes the stoichiometric matrix (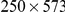). The quantity 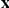 denotes the concentration vector of proteins (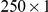). The term 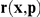 denotes the vector of reaction rates (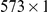). The 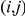 element of the matrix 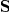, denoted by 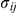, described how protein 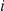 was involved in rate 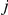. If 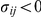, then protein 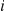 was consumed in 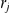. Conversely, if 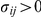, protein 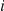 was produced by 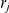. Lastly, if 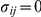, then protein 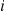 was not involved in rate 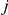. We assumed mass-action kinetics for each interaction in the network. The rate expression for interaction 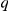 was given by:
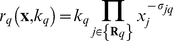
(2)The set 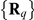 denotes reactants for reaction 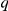 while 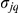 denotes the stoichiometric coefficient (element of the matrix 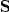) governing species 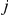 in reaction 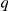. The quantity 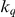 denotes the rate constant governing reaction 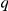. All reversible interactions were split into two irreversible steps. Model equations were generated using UNIVERSAL from an SBML input file (available in the supplemental materials Protocol S1). UNIVERSAL is an open source Objective-C/Java code generator, which is freely available as a Google Code project (http://code.google.com/p/universal-code-generator/). The model equations were solved using the LSODE routine in OCTAVE (v 3.0.5; www.octave.org) on an Apple workstation (Apple, Cupertino, CA; OS X v10.6.4).

When calculating the response of the model to the addition of insulin or other growth factors, we first ran to steady state and then issued the perturbation. The steady state was estimated numerically by repeatedly solving the model equations and estimating the difference between subsequent time points:
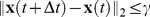
(3)The quantities 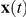 and 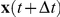 denote the simulated concentration vector at time 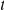 and 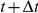, respectively. The 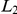 vector-norm was used as the distance metric, where 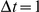 s and 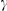 = 0.001 for all simulations.

### Estimation and cross-validation of a population of models using Pareto Optimal Ensemble Techniques (POETs)

We used multiobjective optimization in combination with cross-validation to estimate an ensemble of initiation models. Multiobjective optimization in combination with cross-validation allowed us to address qualitative conflicts in the training data, and to protect against model over-training. While computationally more complex than single-objective formulations, multiobjective optimization is an important tool to address qualitative conflicts in training data that arise from experimental error or cell-line artifacts [76]. Multiobjective optimization balances these conflicts allowing us to identify a consensus model population. In this study we used the Pareto Optimal Ensemble Technique (POETs) to perform the optimization. POETs integrates standard search strategies e.g., Simulated Annealing (SA) or Pattern Search (PS) with a Pareto-rank fitness assignment [52]. Denote a candidate parameter set at iteration 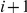 as 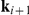. The squared error for 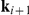 for training set 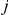 was defined as:
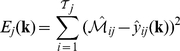
(4)The symbol 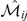 denotes scaled experimental observations (from training set 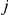) while 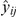 denotes the scaled simulation output (from training set 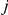). The quantity 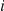 denotes the sampled time-index and 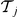 denotes the number of time points for experiment 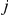. In this study, the experimental data used for model training was typically the band intensity from immunoblots, where intensity was estimated using the ImageJ software package [77]. The scaled measurement for species 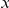 at time 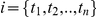 in condition 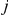 is given by:
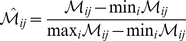
(5)Under this scaling, the lowest intensity band equaled zero while the highest intensity band equaled one. A similar scaling was defined for the simulation output. By doing this scaling, we trained the model on the relative change in blot intensity, over conditions or time (depending upon the experiment). Thus, when using multiple data sets (possibly from different sources) that were qualitatively similar but quantitatively different e.g., slightly different blot intensities over time or condition, we captured the underlying trends in the scaled data.

We computed the Pareto rank of 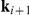 by comparing the simulation error at iteration 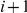 against the simulation archive 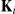. We used the Fonseca and Fleming ranking scheme [78] to estimate the number of parameter sets that dominate 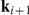. Parameter sets with increasing rank are progressively further away from the optimal trade-off surface. The parameter set 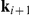 was accepted or rejected by POETs with probability 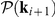:
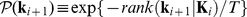
(6)where 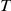 is the annealing temperature and 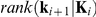 denotes the Pareto rank for 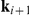. The annealing temperature was discretized into 10 quanta between 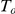 and 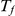 and adjusted according to the schedule 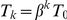 where 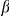 was defined as 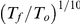. The initial temperature was given by 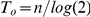, where 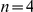 was used in this study and the final temperature was 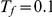. The epoch-counter 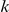 was incremented after the addition of 100 members to the ensemble. Thus, as the ensemble grew, the likelihood of accepting parameter sets with a large Pareto rank decreased. To generate parameter diversity, we randomly perturbed each parameter by 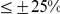. We performed a local pattern search every 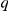 steps to minimize the residual for a single randomly selected objective. The local pattern-search algorithm has been described previously [79].

A leave-three-out cross-validation strategy was used to simultaneously calculate the training and prediction error during the parameter estimation procedure [80]. The 24 training data sets were partitioned into eight subsets, each containing 21 data sets for training and three data sets for validation. The leave-three-out scheme generated 18,886 probable models. From the approximately 6000 rank zero models, we iteratively selected 50 random models from each cross-validation trial with the lowest correlation and shortest Euclidian distance to the origin (minimum error). This selection technique produced sub-ensembles with low set-to-set correlation (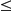
0.50) and minimum training error.

### Sensitivity and robustness analysis of the initiation model population

Sensitivity coefficients were calculated for 40 models selected from the ensemble (rank-zero, low-correlation, minimum error selection). First-order sensitivity coefficients at time 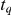:
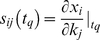
(7)were computed by solving the kinetic-sensitivity equations [81]:
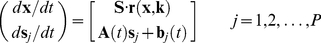
(8)subject to the initial condition 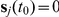. The quantity 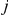 denotes the parameter index, 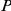 denotes the number of parameters in the model, 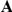 denotes the Jacobian matrix, and 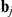 denotes the 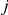
th column of the matrix of first-derivatives of the mass balances with respect to the parameters. Sensitivity coefficients were calculated by repeatedly solving the extended kinetic-sensitivity system for forty parameters sets selected from the final 400 member ensemble. These sets were chosen to be comparable to the final 400 member ensemble on the basis of parametric coefficient of variation (CV); the sets selected for sensitivity analysis had a mean CV of 0.85
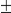
0.5 and a mean correlation of approximately 0.6. Thus, there were diverse and uncorrelated. The Jacobian 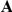 and the 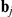 vector were calculated at each time step using their analytical expressions generated by UNIVERSAL.

The resulting sensitivity coefficients were scaled and time-averaged (Trapezoid rule):
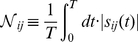
(9)where 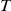 denotes the final simulation time. The time-averaged sensitivity coefficients were then organized into an array for each ensemble member:
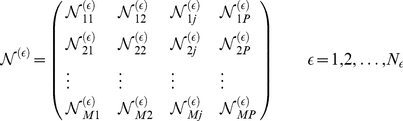
(10)where 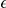 denotes the index of the ensemble member, 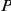 denotes the number of parameters, 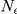 denotes the number of ensemble samples and 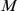 denotes the number of model species. To estimate the relative fragility or robustness of species and reactions in the network, we decomposed the 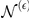 matrix using Singular Value Decomposition (SVD):
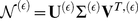
(11)Coefficients of the left (right) singular vectors corresponding to largest 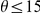 singular values of 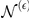 were rank-ordered to estimate important species (reaction) combinations. Only coefficients with magnitude greater than a threshold (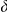 = 0.001) were considered. The fraction of the 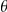 vectors in which a reaction or species index occurred was used to determine its importance (sensitivity ranking). The sensitivity ranking was compared between different conditions to understand how control in the network shifted as a function of perturbation or time (Fig. 5).

Robustness coefficients were calculated as shown previously [60]. Robustness coefficients (denoted by 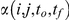) are the ratio of the integrated concentration of a network marker in the presence (numerator) and absence (denominator) of a structural or operational perturbation. The quantities 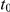 and 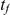 denote the initial and final simulation time, respectively, while 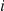 and 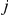 denote the indices for the marker and the perturbation respectively. If 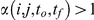, then the perturbation *increased* the marker concentration. Conversely, if 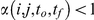 the perturbation *decreased* the marker concentration. Lastly, if 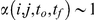 the perturbation did not influence the marker concentration. Robustness coefficients were calculated over 400 models selected from the ensemble (rank-zero, low-correlation, minimum error selection). Convergence analysis suggested that the qualitative conclusions drawn from the robustness analysis would not change if more than N = 400 parameter sets were sampled (Fig. S1).

## Supporting Information

Figure S1 **Effect of the ensemble size on the knockdown simulations.** Fold change of the translational activity was calculated for ensemble sizes of N = 50 (white fill), N = 100 (light grey), N = 200 (dark grey) and N = 400 (black) randomly selected parameter sets in the presence and absence of insulin. For the majority of the perturbations, the robustness coefficients converged for as few as 50 parameter sets. In a small number of other cases, the robustness coefficients varied significantly up to 200 parameter sets. Between 200–400 sets the robustness coefficients largely converged to qualitatively and quantitatively similar answers.
(EPS)

Protocol S1 **Supporting simulation protocols.** Protocol S1 file includes SBML file of the network used with nominal rate constants and initial conditions and ensemble of parameter files generated by POETs and used for model analysis. Further details of each file is included in a README file included in the zip.
(TAR.BZ2)
